# Ellagic acid effects on disease severity, levels of cytokines and T-bet, RORγt, and GATA3 genes expression in multiple sclerosis patients: a multicentral-triple blind randomized clinical trial

**DOI:** 10.3389/fnut.2023.1238846

**Published:** 2023-09-19

**Authors:** Sahar Jafari Karegar, Naheed Aryaeian, Ghazaleh Hajiluian, Katsuhiko Suzuki, Farzad Shidfar, Masoud Salehi, Bahram Haghi Ashtiani, Pooya Farhangnia, Ali-Akbar Delbandi

**Affiliations:** ^1^Department of Nutrition, School of Public Health, Iran University of Medical Sciences, Tehran, Iran; ^2^Faculty of Sport Sciences, Waseda University, Tokorozawa, Japan; ^3^Department of Statistics, School of Public Health, Iran University of Medical Sciences, Tehran, Iran; ^4^Firouzgar Hospital, Iran University of Medical Sciences, Tehran, Iran; ^5^Department of Immunology, School of Medicine, Iran University of Medical Sciences, Tehran, Iran; ^6^Immunology Research Center, Institute of Immunology and Infectious Disease, Iran University of Medical Sciences, Tehran, Iran

**Keywords:** multiple sclerosis, ellagic acid, pathogenesis, inflammation, disease severity

## Abstract

**Background:**

Multiple sclerosis (MS) is a chronic autoimmune disease. Ellagic acid is a natural polyphenol and affects the fate of neurons through its anti-inflammatory and antioxidant properties. The present study aimed to investigate ellagic acid effects on disease severity, the expression of involved genes in the pathogenesis of MS, and the levels of related cytokines.

**Methods:**

The present study was a triple-blind clinical trial. Eligible patients were randomly assigned to two groups: Ellagic acid (25 subjects) for 12 weeks, receiving 180 mg of Ellagic acid (Axenic, Australia) and the control group (25 subjects) receiving a placebo, before the main meals. Before and after the study, the data including general information, foods intake, physical activity, anthropometric data, expanded disability status scale (EDSS), general health questionnaire (GHQ) and pain rating index (PRI), fatigue severity scale (FSS) were assessed, as well as serum levels of interferon-gamma (IFNγ), interleukin-17 (IL-17), interleukin-4 (IL-4) and transforming growth factor-beta (TGF-β), nitric-oxide (NO) using enzyme-linked immunoassay (ELISA) method and expression of T-box transcription factor (Tbet), GATA Binding Protein 3 (GATA3), retinoic acid-related orphan receptor-γt (RORγt) and Glyceraldehyde-3-phosphate dehydrogenase (GAPDH) genes were determined using Real-Time Quantitative Reverse Transcription PCR (RT-qPCR) method.

**Findings:**

Ellagic acid supplementation led to a reduction in IFNγ, IL-17, NO and increased IL-4 in the ellagic acid group, however in the placebo group no such changes were observed (−24.52 ± 3.79 vs. -0.05 ± 0.02, *p* < 0.01; −5.37 ± 0.92 vs. 2.03 ± 1.03, *p* < 0.01; −18.03 ± 1.02 vs. -0.06 ± 0.05, *p* < 0.01, 14.69 ± 0.47 vs. -0.09 ± 0.14, *p* < 0.01, respectively). Ellagic acid supplementation had no effect on TGF-β in any of the study groups (*p* > 0.05). Also, the Tbet and RORγt genes expression decreased, and the GATA3 gene expression in the group receiving ellagic acid compared to control group significantly increased (0.52 ± 0.29 vs. 1.51 ± 0.18, *p* < 0.01, 0.49 ± 0.18 vs. 1.38 ± 0.14, *p* < 0.01, 1.71 ± 0.39 vs. 0.27 ± 0.10, *p* < 0.01). Also, ellagic acid supplementation led to significant decrease in EDSS, FSS and GHQ scores (*p* < 0.05), and no significant changes observed in PRI score (*p* > 0.05).

**Conclusion:**

Ellagic acid supplementation can improve the health status of MS patients by reduction of the inflammatory cytokines and Tbet and RORγt gene expression, and increment of anti-inflammatory cytokines and GATA3 gene expression.

**Clinical trial registration**: (https://en.irct.ir/trial/53020), IRCT20120415009472N22.

## 1. Introduction

Multiple sclerosis (MS) is an autoimmune disease that leads to a gradual damage and loss of the myelin sheath of neurons in the spinal cord, brain and optic nerve (1). These injuries then lead to atrophy of the affected nerves over time. The atrophy that occurs at the onset of the disease is mild but progresses over time, eventually leading to numerous disabilities in these patients (1). Among the Middle Eastern countries, Iran has the highest prevalence rate of MS (2). The onset of the disease usually occurs in early to middle adulthood, between 20 and 40 years old, and the prevalence is higher in women (3).

MS disease has different forms with varying severity. The five main types of MS are relapsing–remitting (RR), progressive-remitting (PR), progressive-remitting (RP), primary progressive (PP), and secondary progressive (SP) MS. In approximately 85% of MS patients, the RR phase occurs first and then the SP phase (4). The RR type is the most common form of MS, in which inflammatory attacks on myelin and nerve fibers lead to deterioration of nerve function. In the RR type, symptoms vary from one patient to another, sometimes intensifying unexpectedly (this is called relapse and exacerbation) and then subsiding. The RR phase involves T-helper 1 (Th1) and Th17 cells that invade the central nervous system (CNS), and the SP phase results from inflammation caused by activation of innate immunity (5).

Th1 and Th17 cells play the main role in the pathophysiology of MS, and the inflammatory cytokines they produce lead to an increase in the permeability of the blood–brain barrier (BBB) to monocytes and macrophages (6). Th1 cells are actively present in the bloodstream of MS patients. These cells are also present in the damaged parts of the CNS and cause the production of inflammatory cytokines, including interferon-gamma (IFNγ) (7). The differentiation of immature T cells is under the influence of transcription factors that affect the expression of cytokine genes in these cells. T-bet is a transcription factor of the T-box transcription factor family that causes differentiation of immature T cells into Th1 cells and prevents differentiation of immature T cells into Th2 cells. The activation of T-bet is also the result of the action of IFNγ and IL-12, and after this activation, the number of Th1 cells and cytokines increases (8, 9). The conversion of immature T cells into Th17 cells is influenced by signal transducer and activator of transcription-3 (STAT3), and retinoic acid-related orphan receptor-γt (RORγt). Th17 cells adhere to the BBB (10), and their major cytokine, IL-17, increases BBB permeability and induces neutrophil transfer to the CNS. As a result, antigen (Ag)-specific CD4+ and CD8+ T cells are secreted into the CNS. The produced T cells pass through the BBB *via* the immune pathway and form the basis for the passage of monocytes into the CNS. Following this process, remnant microglia and astrocytes are activated, antigen-specific Th cells are differentiated, and the release of inflammatory cytokines leads to axon damage and loss (11, 12).

Th2 and Treg cells, which produce anti-inflammatory cytokines, play a modulatory and protective role against MS progression. To differentiate immature T cells into Th2 cells, IL-4 affects and activates the Th2 transcription factor (GATA3). As a result, the concentration of Th2 cytokines, including IL-4, increases (12, 13). Many drugs, including glatiramer acetate, reduce relapse in MS patients by altering the differentiation of immature T cells toward Th2 production and increasing IL-4 levels and inhibiting IFNγ secretion. This shows that increasing anti-inflammatory cytokines has a positive effect on the healing process of MS patients (13–15). Treg cells include a subset of CD4+ T lymphocytes that have immunoregulatory effects due to their ability to inhibit Th1 and Th17 cells. Treg cells can protect a person from autoimmune diseases. This is because CD4+ Treg cells inhibit inflammatory processes, and their cytokines, such as TGF-β, are considered a therapeutic target in MS patients (16). IL-10 and TGF-β are regulatory cytokines whose function affects Treg cell differentiation. IL-10 inhibits the secretion of Th1 cytokines and the progression of MS (17). Thus, the mice whose IL-10 gene was knocked out had a higher susceptibility to the development of MS, and in contrast, the mice whose expression of the IL-10 gene was overexpressed showed resistance to the development of MS (18).

Therapeutic strategies that shift immune system responses from Th1 to Th2 and Th17 to regulatory T cells may be effective in treating MS. Drugs approved by the Food and Drug Administration (FDA) for MS patients include beta-interferon, glatiramer acetate (GA), mitoxantrone, and natalizumab. All of these drugs can only affect MS disease to some degree, suggesting that more effective ways need to be found to affect disease progression as soon as possible (19, 20). Inflammation and apoptosis have been shown to have detrimental effects on brain cell function, and natural antioxidants play an important protective role in controlling this process (21).

Ellagic acid is a polyphenolic lactone found in a variety of vegetables and fruits, including pomegranates, strawberries, eucalyptus leaves, green tea, raspberries, and blackberries (22). The most important polyphenol in pomegranate is punicalagin, which is not absorbed in its healthy and intact form in the intestine but can be hydrolyzed and converted to ellagic acid. When ellagic acid is orally ingested, it is converted by the intestinal microbiota under the influence of a specific metabolism into urolithins, which are much better absorbed in the digestive tract (22–24). Ellagic acid is a natural tannic acid derivative and influences the fate of neurons through its anti-inflammatory (25), antioxidant (26), and antidepressant effects (27). The limited pharmacological data on ellagic acid indicate that its serum elimination half-life in humans is 8.4 ± 1.4 h (200 ng/mL, oral). Serum elimination was also rapid when taken orally in animal studies (28). Previous studies have shown that ellagic acid reduces the inflammatory response in animal models of colitis (29), acute lung injury (30), and acute inflammation (31). Thus, treatment with ellagic acid leads to a reduction in the level of IL-17, IFNγ, and suppression of inflammatory cytokines (32, 33). in animal studies, ellagic acid has been shown to lead to an increase in some anti-inflammatory cytokines, including IL-4 (33, 34). Some experimental studies have also shown that ellagic acid decreases BBB permeability and TNF-α levels in the CNS (35). In clinical trials, the highest dose studied was 180 mg per day, and no side effects were reported (36). In addition, some studies have shown the neuroprotective effects of ellagic acid (37). Therefore, the aim of the present study was to investigate the effect of ellagic acid on disease severity, MS patienst disability status, the expression of genes involved in the pathogenesis of MS, the levels of associated inflammatory cytokines and oxidative stress in these patients.

## 2. Methods

### 2.1. Type of the study and participants

The present study was a triple-blind, multicentral, placebo-controlled clinical trial conducted in patients with MS. The population study is patients with MS, who were referred to Firouzgar and Hazrate Rasoule Akram hospitals (Tehran, Iran). Patients with MS of either sex who met the criteria for participation in the study and agreed to cooperate were enrolled in the study under the supervision of a neurologist after confirming the disease.

The inclusion criteria for participation in the present study were as follows: MS confirmation getting based on McDonald criteria (38) and magnetic resonance imaging (MRI) by neurologist, clinical status of relapse-remittance based on the criteria proposed by Lublin and Reingold (39), age between 18 and 55 years and EDSS score of less than 5.5.

**Figure 1:**
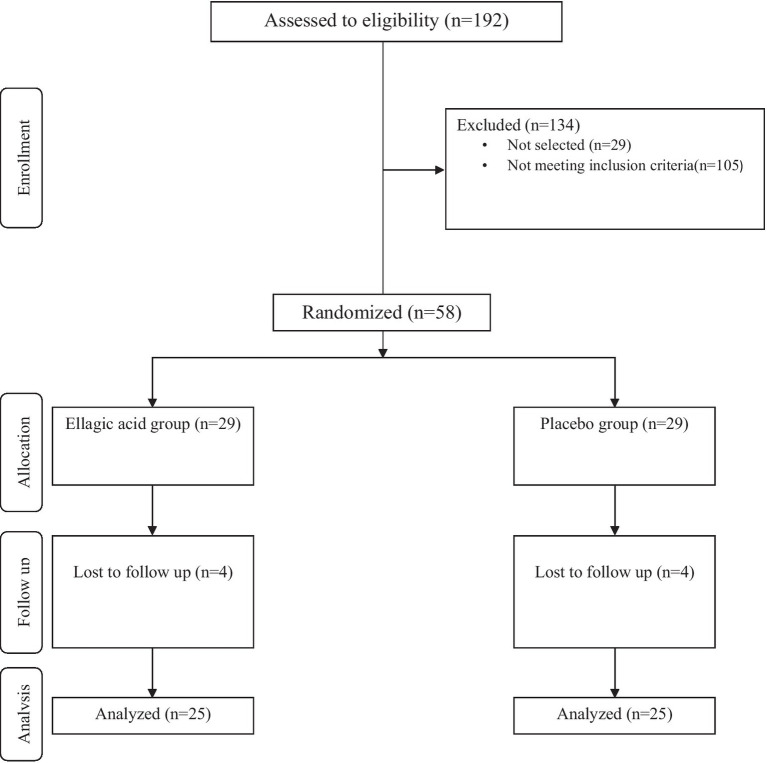
Summary of participants recruitment based on the consolidated standards of reporting trials (CONSORT) flow diagram.

The exclusion criteria were: refusal to continue participation in present study, change in the severity of the disease during the study, relapse occurrence during the intervention period, changes in dosage and type of medication consumed during the study, changes in the physical activity of the patients, no use of less than 90% ellagic acid supplements, ellagic acid or other supplements usage within1 month before the start of the study and within the study, estrogen, progesterone, diuretics, and corticosteroids within 1 month prior to the study, suffering from autoimmune disease, and pregnancy or breastfeeding. Also, patients with history of allergy and smokers excluded. The study flow diagram including different stages of study are presented in Figure 1.

The study protocol was approved by Ethics Committee of Iran University of Medical Sciences (Ethics code: IR.IUMS.REC.1399.1000). The study protocol was registered on website of the Iranian Registry of Clinical Trials (identifier: IRCT20120415009472N22, at the date of 19/12/2020). Written informed consent was provided from the participants.

### 2.2. Sample size calculation and

In this study, in order to determine the number of required patients, according to type I error equal to 5%, a power of 90% and EDSS as one the primary outcomes, the standard deviation for the EDSS was considered for calculation (40). Considering the 20% possibility for sample dropout, the volume of studied patients is estimated to be 29 people in each group and 58 people in total.

### 2.3. Sampling, blinding and randomization

Sampling was done by convenience sampling method. The selected patients based on the inclusion criteria were randomly assigned to two groups receiving ellagic acid and placebo. The method of random allocation was done by the balanced block method. None of the patients and the interviewer and analysis consultant knew the sample in which the group were placed (triple-blind randomized trial). The manufacturer was responsible for blinding the supplements by coding.

Then, a total of 58 MS patients were randomly assigned to ellagic acid (*n* = 29) and placebo (*n* = 29) groups. Each ellagic acid capsules (90 mg) and placebo capsules (maltodextrin) were taken twice a day by MS patients in intervention and control groups, respectively. Ellagic acid and placebo capsules were similar in shape, weight, taste, size, odor and color and produced by Axenic Company, Australia. Ellagic acid purity was 99.9% and the dosage of ellagic acid was chosen based on previous studies (37).

Patients were given packets of ellagic acid or placebo sufficient for 4 weeks of consumption in the order they entered the study, and at the fourth and eighth weeks they were again given supplements or placebo. They were told to take two capsules of ellagic acid or placebo daily after lunch, and this process continued for 12 weeks. At each visit, patients were asked to bring the packet of supplements or placebo. If patients did not consume less than 90% of the supplements or placebo at each of the four-, eight-, or 12-week visits, they were excluded from the study.

During the study, patients were reminded to take the supplements by phone call and text message. During the intervention period, patients were asked not to change their diet or physical activity and not to take any dietary supplements without the advice of their treating physician. They were also asked to inform us of any change in the dosage of their medications during the study period.

### 2.4. Questionnaires

In the phase before the start of the intervention, general characteristics including age, sex, duration of disease, and dosage of medication taken by participants were recorded. To assessment of the patients’ food intake, three 24-h food intake questionnaire was completed on the first, sixth and twelfth weeks of the study (two normal days and one day off) and analyzed by Nutritionist 4 software (USA). In addition, the International Physical Activity Questionnaire (IPAQ) was completed to check the level of physical activity as a confounding factor before and after the intervention. The anthropometrical questionnaire is included height and weight. Height measurement was performed using a strip meter with a precision of 0.1 cm and weight measurement, using a digital scale of 100 grams (Seca, Germany) under standard conditions. Also, the body mass index was calculated through the formula. GHQ questionnaire consisting of 28 questions were used to assess the general health status (41). The McGill Pain Questionnaire was also used to calculate the pain rating index (PRI) score, which has 78 items and 20 groups that examine the different dimensions of pain (42). Fatigue severity scale (FSS) questionnaire was used to assess the fatigue severity in MS patients (43). All questionnaires validity and reliability were approved in Iranian population in recent studies (41–43).

### 2.5. Immunological assessments

Blood samples were drawn from the patients at the pre- and post-intervention and centrifuged at a speed of 2000 rpm for 10 min until the serum was separated. Then, the serum of the samples for measuring the indices of interleukin 4, interleukin 17, TGF-β, and IFNγ were kept in a freezer at-80°C until assay. The levels of IL-4 (Zellbio, Germany), IL-17 (Zellbio, Germany), TGF-β (Zellbio, Germany), IFNγ (R&D Systems, USA), and nitric oxide (NO) (Zellbio, Germany) were measured using enzyme-linked immunoassay (ELISA) kits based on the instructions in the kit guidelines. The protocol of the measurement method was as follows: First, the reagents, samples, and standards were prepared according to the instructions. Then, 100 microliters of the standard solution and sample were added to each well of the 96-well plate and incubated at 37°C for 2 h, and the liquid in each well was drained. Then, 100 microliters of biotin antibody (x1) were added to each well and incubated at 37°C for 1 h. Then, the wells were emptied and washed three times with PBS solution (phosphate-buffered saline). Then, 100 microliters of HRP-avidin (x1) were added to each well and incubated at 37°C for 1 h. Again, the wells were emptied and washed 5 times with PBS solution. Then, 90 microliters of TMB (tetramethylbenzidine) substrate were added to each well and incubated at 37°C (in the dark) for 30 min. Finally, 50 microliters of the stop solution were added to each well and the light absorbance (OD) of the samples was evaluated within 5 min using an ELISA reader (Hyperion, MPR4++, USA) at a wavelength of 450 nm.

### 2.6. Gene expression assessment

The expression of Tbet, RORγ, GATA3, and GAPDH genes was also measured using the real-time Quantitative Reverse Transcription (RT-qPCR) method. Peripheral blood mononuclear cells (PBMCs) were first isolated to assess gene expression. Ficoll and concentration gradients were used to isolate PBMCs. To isolate PBMCs, 10 mL of peripheral blood was first poured into a heparin-containing tube and the same volume of PBS was added at a temperature of 4°C. Mixing was performed with slow and circular movements. In a separate tube, 3 mL of Ficoll was added and half of the diluted blood was added to the Ficoll in the tube. After the addition of blood, the tube was placed in a refrigerated centrifuge (temperature 4°C) and centrifuged at 800 g for 40 min. After centrifugation, four different layers, including plasma, PBMC, Ficoll, and red blood cells, were observed from top to bottom. The PBMC layer was separated from each tube and poured into a 50 mL tube. Then, up to 40 mL of PBS solution was added and placed in a refrigerated centrifuge (temperature 4°C) and centrifuged at a speed of 600× *g* for 10 min. After centrifugation, the supernatant was discarded. Then, 2 mL of PBS solution was added to the cells located at the bottom of the tube. After adding the PBS solution, the cell layer was dissolved and homogenized by pipetting in the solution. Finally, the homogenized solution was transferred to a 1.5 mL microtube and centrifuged again (4°C at 600× *g* speed, 10 min). After centrifugation, the supernatant was discarded, and the PBMCs were simultaneously used for RNA extraction. RNA extraction was performed using the Rneasy plus mini kit (Qiagen, Germany) according to the kit instructions. This step was performed with nuclease-free equipment under a hood disinfected with alcohol and sterilized with UV light. A Nano Drop device (Thermo Scientific, USA) was used to determine the purity of RNA, and the absorbance ratio of 260/280, 1.8–2.2 was considered high purity based on the kit protocol. RNA concentration was also determined using nanodrops. The extracted RNA was stored in a freezer at-80°C until cDNA synthesis. For cDNA synthesis, 500 ng of RNA was used with the Quantitect reverse transcriptase commercial kit from Qiagen (Qiagen, Germany). Then, the prepared cDNA was stored in the freezer at-20°C until gene expression was measured. Subsequently, the synthesised cDNAs were stored in a freezer at-20°C until the time of gene expression measurement. To select the appropriate primer, the sequence of the primers was taken from reliable articles that similarly investigated the expression of the genes in this study in PBMC, and the properties of the primers were checked using Gene Runner software. The website www.ensembl.org was used to check the sequence of the desired genes and also whether the forward and reverse primers were located in two exons. The site http://www.ncbi.nlm.nih.gov/tools/primer-blast was also used to control the specificity of the primers. For the GAPDH gene (housekeeping gene), primers were selected from the studies in the same way (Table 1). After primer preparation, gene expression was measured using the Rotor-Gene Q (Qiagen, Hilden, Germany) instrument and the SYBR Green method. All experiments were performed with nuclease-free devices under a hood disinfected with alcohol and sterilized with ultraviolet (UV) light. Real-time PCR analysis was done by fold change calculation based on 2^–∆∆Ct^.

**Table 1:** Primers used for quantitative real-time PCR analysis.

Gene	Type	Sequence
GATA3	Forward	5′- ACCACAACCACACTCTGGAGG A-3′
	Reverse	5′- TCGGTTTCTGGTCTGGATGCC T-3′
RORγt	Forward	5′- GCCAAGGCCGGCAGAGCCAA-3′
	Reverse	5′- AAGAAGCCCTTGCACCCCTCACA-3′
Tbet	Forward	5′- CCACCTGTTGTGGTCCAAGT −3′
	Reverse	5′- AACATCCTGTAGTGGCTGGTG-3′
GAPDH	Forward	5′-GCACCGTCAAGGCTGAGAAC-3′
	Reverse	5′-TGGTGAAGACGCCAGTGGA-3′

### 2.7. Data analysis

Data analysis was performed using SPSS version 24 software. Descriptive statistics methods including frequency distribution tables and central and dispersion indices were used to describe the samples. The Kolomogorov-Smirnov test was performed to determine distribution of variables. Levene’s test was used to determine equality of variances.

In this study, to compare the quantitative variables at baseline and to compare the average changes in these variables during the study between groups, the independent t test was used. In addition, to compare the quantitative variables within each group before and after the intervention, the paired t test was used. If there was a confounder variable, covariance analysis was used. Quantitative variables were reported as mean (standard deviation) and 5% was considered as a significant level.

## 3. Results

### 3.1. Baseline characteristics of study participants

Among the 192 people with MS referred to the neurology clinics of Rasool Akram Hospital and the MS Clinic of Firozgar Hospital (Tehran, Iran) from January 2019 to the end of September 1,400, 58 patients with MS were eligible to enter the study, in order to perform Intervention were invited. Based on the Stratified Permuted Block Randomization method, patients were assigned into two groups: (1) group receiving ellagic acid supplement (180 mg per day), (2) control group receiving placebo containing maltodextrin. In the second visit of the patients in the middle of the intervention, 3 patients in the group receiving ellagic acid (3 women) and 4 patients in the group receiving placebo (4 women) were excluded from the study due to changes in the course of the disease and the drugs received. In the last visit, one patient in the group receiving ellagic acid was excluded from the study due to a change in the medications received. Thus, in both groups, 25 patients entered the final stage and data analysis (Figure 1).

The rate of patient compliance with the intervention was 92.23 and 92.42% in the ellagic acid and placebo groups, respectively.

The baseline characteristics of the subjects in the present study are provided in Table 2. In both groups, 12% of the participants were male and 88% were female. The average age of people in the ellagic acid group was 42.89 ± 9.48 and in the control group it was 37.98 ± 9.02, which were statistically significantly different from each other. Also, there were significant difference between two study groups regarding the disease duration (*p* < 0.05). The patients in the present study were not significantly different in terms of the EDSS score, and the drugs used (Table 2).

**Table 2:** Baseline characteristics of participants in ellagic acid and control groups.

Variable		Total (*n* = 50)	Ellagic acid (*n* = 25)	Control (*n* = 25)	*p*-value^a^
Age (years)		39.51 ± 9.15	42.89 ± 9.48	37.98 ± 9.02	0.047
Height (cm)		164.88 ± 8.22	164.29 ± 7.93	165.02 ± 8.44	0.539
Gender Number (%)	Male	6(12)	3(12)	3(12)	–
	Female	44(88)	22(88)	22(88)	–
Weight (kg)		68.51 ± 12.37	69.10 ± 12.52	67.93 ± 12.58	0.482
BMI (kg/m2)		25.51 ± 3.37	25.42 ± 3.52	25.38 ± 3.29	0.891
MS duration (years)		5.27 ± 0.54	4.42 ± 0.61	6.18 ± 0.49	0.013
Physical activity (MET-h/week)		33.48 ± 5.12	32.99 ± 5.26	33.89 ± 5.02	0.274
EDSS score		2.59 ± 0.35	2.60 ± 0.38	2.58 ± 0.31	0.750
MS drugs Number (%)	Resigen	6(12)	3(12)	3(12)	–
	CinnoVex	38(76)	19(76)	19(76)	–
	Betaferon	6(12)	3(12)	3(12)	–

Data are presented as mean ± SD for quantitative and frequency (%) for qualitative variables.

BMI, body mass index; EDSS, Expanded Disability Status Scale.

^a^Independent Sample *t*-test.

*p* value < 0.05 considered significant.

### 3.2. Effect of ellagic acid supplementation on dietary intake

In Table 3, the findings related to energy, carbohydrate, fat, protein, fiber, and micronutrients has been shown. There were no significant differences between the two groups regarding any of the findings of energy, macronutrients, fiber and micronutrients at baseline and end of the intervention (*p* > 0.05). Also, there were no significant differences regarding dietary intake within ellagic acid and control groups at the end of the study compared to their respective baselines (*p* > 0.05).

**Table 3:** Dietary intake of participants in ellagic acid and control groups at weeks 0, 6 and 12 of the intervention.

Variable	Time	Ellagic acid group (*n* = 25)	Control (*n* = 25)	Mean differences (95% CI)	*p*-value^a^
Energy (Kcal)	Week 0	2214.00 ± 426.61	2134.50 ± 293.73	80.92 (−10, 177)	0.18
	Week 6	2202.00 ± 389.31	2109.50 ± 269.41	93.55 (−9, 158)	0.28
	Week 12	2182.00 ± 408.01	2124.31 ± 124.58	58.04 (−21,92)	0.13
	*p*-value^b^	0.06	0.09		
Carbohydrate (g/d)	Week 0	317.15 ± 46.33	320.00 ± 41.31	−3.12 (−4.18, 5.27)	0.82
	Week 6	303.40 ± 37.80	309.60 ± 40.95	−6.2 (−10.63, 9.82)	0.74
	Week 12	323.00 ± 29.70	325.18 ± 57.69	−1.81 (−3.46, 5.39)	0.19
	*p*-value^b^	0.86	0.74		
Protein (g/d)	Week 0	70.30 ± 34.76	70.85 ± 18.64	−0.55 (−1.77, 1.42)	0.91
	Week 6	71.65 ± 28.98	69.40 ± 18.27	2.25 (−0.67, 3.09)	0.82
	Week 12	69.95 ± 27.12	71.25 ± 17.20	−1.31 (−2.55, 1.40)	0.06
	*p*-value^b^	0.38	0.06		
Total fat (g/d)	Week 0	84.60 ± 17.99	83.85 ± 9.10	0.75 (−0.08, 1.17)	0.76
	Week 6	80.25 ± 14.25	81.30 ± 14.25	−1.15 (−2.45, 1.32)	0.49
	Week 12	84.81 ± 18.17	84.00 ± 10.81	0.81 (−0.94, 1.42)	0.09
	*p*-value^b^	0.45	0.14		
SAFA (g/d)	Week 0	22.98 ± 6.34	20.00 ± 4.12	2.98 (−1.57, 3.93)	0.18
	Week 6	19.34 ± 3.91	22.29 ± 5.44	−2.95 (−5.48, 3.28)	0.28
	Week 12	21.47 ± 2.11	21.94 ± 4.85	−0.47 (−1.55, 0.39)	0.13
	*p*-value^b^	0.34	0.17		
MUFA (g/d)	Week 0	19.75 ± 4.33	16.08 ± 2.38	3.67 (−2.75, 5.21)	0.82
	Week 6	18.33 ± 4.57	18.72 ± 4.29	−0.39 (−1.15, 1.44)	0.74
	Week 12	19.81 ± 5.15	18.66 ± 4.62	1.15 (−0.28, 2.19)	0.19
	*p*-value^b^	0.98	0.25		
PUFA (g/d)	Week 0	11.10 ± 2.42	11.39 ± 1.67	−0.19 (−0.78, 0.83)	0.91
	Week 6	11.11 ± 1.34	11.57 ± 2.23	−0.46 (−0.99, 1.12)	0.82
	Week 12	11.08 ± 2.07	11.44 ± 2.17	−0.36 (−1.02, 0.82)	0.06
	*p*-value^b^	0.27	0.09		
Vitamin A (mg)	Week 0	415.25 ± 66.74	390.85 ± 48.69	24.47 (−11, 39)	0.76
	Week 6	386.15 ± 51.46	397.85 ± 62.35	−11.7 (−17, 23)	0.49
	Week 12	367.27 ± 45.34	400.21 ± 51.47	−32.94 (−52, 101)	0.09
	*p*-value^b^	0.07	0.09		
Vitamin E (mg)	Week 0	4.41 ± 0.89	4.89 ± 0.80	−0.48 (−1.26, 1.73)	0.17
	Week 6	4.89 ± 0.80	4.67 ± 0.93	0.22 (−0.18, 0.67)	0.29
	Week 12	4.47 ± 0.99	4.80 ± 0.12	−0.33 (−0.92, 1.15)	0.10
	*p*-value^b^	0.63	0.08		
Vitamin D (μg)	Week 0	5.15 ± 0.51	4.81 ± 0.70	0.34 (−0.16, 0.91)	0.81
	Week 6	5.00 ± 0.62	4.82 ± 0.67	0.18 (−0.27, 0.59)	0.58
	Week 12	5.08 ± 0.81	4.20 ± 0.90	0.88 (−0.08, 1.12)	0.92
	*p*-value^b^	0.58	0.25		
Vitamin C (mg)	Week 0	17.75 ± 3.70	16.84 ± 3.03	0.91 (−0.22, 1.70)	0.77
	Week 6	16.72 ± 3.17	16.19 ± 2.76	0.53 (−0.11, 0.92)	0.93
	Week 12	17.28 ± 1.18	16.96 ± 2.55	0.32 (−0.62, 1.39)	0.07
	*p*-value^b^	0.45	0.07		
Calcium (mg)	Week 0	1056.30 ± 167.68	892.20 ± 168.80	164 (−83, 226)	0.08
	Week 6	1019.00 ± 127.36	917.50 ± 174.85	102 (−107, 261)	0.59
	Week 12	1087.01 ± 133.36	908.63 ± 185.25	179 (−114, 293)	0.32
	*p*-value^b^	0.08	0.59		
Iron (mg)	Week 0	13.56 ± 2.69	10.85 ± 2.38	2.71 (−1.43, 3.82)	0.12
	Week 6	10.90 ± 2.10	12.60 ± 2.49	−1.70 (−3.71, 2.99)	0.92
	Week 12	10.74 ± 10.54	11.91 ± 1.24	−1.17 (−4.12, 2.31)	0.06
	*p*-value^b^	0.06	0.25		
Selenium (μg)	Week 0	0.05 ± 0.01	0.04 ± 0.00	0.01 (−0.02, 0.04)	0.77
	Week 6	0.04 ± 0.01	0.04 ± 0.00	0 (−0.01, 0.03)	0.73
	Week 12	0.04 ± 0.18	0.04 ± 0.01	0 (−0.01, 0.03)	0.09
	*p*-value^b^	0.09	0.39		
Zinc (μg)	Week 0	7.78 ± 1.15	6.01 ± 1.00	1.77 (−0.23, 2.18)	0.37
	Week 6	7.31 ± 1.09	6.06 ± 1.09	1.25 (−1.15, 2.19)	0.45
	Week 12	7.97 ± 1.85	6.04 ± 1.02	1.93 (−1.81, 3.32)	0.06
	*p*-value^b^	0.12	0.81		
Fiber (g/d)	Week 0	15.70 ± 2.60	15.19 ± 2.80	0.51 (−0.77, 1.42)	0.52
	Week 6	14.37 ± 2.50	15.17 ± 3.18	−0.80 (−1.83, 2.49)	0.88
	Week 12	15.98 ± 1.84	15.22 ± 3.84	0.76 (−0.75. 1.88)	0.15
	*p*-value^b^	0.74	0.89		

Data are presented as mean ± SD.

^a^Independent sample *t*-test.

^b^Value of *p* for repeated measures ANOVA performed to assess variations in dietary intakes across periods.

*p* value < 0.05 considered significant.

### 3.3. Effect of ellagic acid supplementation on anthropometric measurements and physical activity

The findings of anthropometric measurements showed no significant differences between two groups regarding weight, BMI, WC and physical activity at baseline and end of the intervention (*p* > 0.05). Also, there were no significant differences regarding weight, BMI, WC and physical activity within ellagic acid and control groups at the end of the study compared to their respective baselines (*p* > 0.05; Table 4).

**Table 4:** Anthropometric measurements and physical activity status in ellagic acid and control groups in the beginning and at the end of the study.

Variable	Time	Ellagic acid group (*n* = 25)	Control (*n* = 25)	Mean differences (95% CI)	*p*-value^a^
Weight (Kg)	Before	69.10 ± 12.52	67.93 ± 12.58	1.17 (−0.52, 1.93)	0.482
	After	69.54 ± 11.32	68.41 ± 12.62	1.12 (−0.88, 1.79)	0.065
	Mean change	0.43 ± 0.09	0.46 ± 0.02	−0.03 (−0.08, 0.05)	0.931
	*p*-value^b^	0.778	0.591		
BMI (Kg/m^2^)	Before	25.42 ± 3.52	25.38 ± 3.29	0.13 (−0.01, 0.28)	0.891
	After	25.41 ± 3.53	25.40 ± 3.27	0.11 (−0.01, 0.32)	0.938
	Mean change	0.01 ± 0.02	0.02 ± 0.01	−0.01 (−0.09, 0.07)	0.289
	*p*-value^b^	0.089	0.142		
Waist circumference (cm)	Before	89.44 ± 6.86	90.16 ± 6.85	−0.75 (−1.21, 1.77)	0.184
	After	89.09 ± 6.83	90.24 ± 6.92	−1.15 (−2.33, 2.91)	0.059
	Mean change	−0.35 ± 0.04	0.08 ± 0.04	−0.44 (−1.04, 1.25)	0.184
	*p*-value^b^	0.312	0.571		
Physical activity (MET-min/week)	Before	32.99 ± 5.26	33.89 ± 5.02	0.10 (−0.01, 0.23)	0.274
	After	32.82 ± 5.35	33.93 ± 5.12	−0.11 (−0.23, 0.01)	0.063
	Mean change	−0.17 ± 0.08	0.04 ± 0.09	−0.21 (−0.42, 0.29)	0.081
	*p*-value^b^	0.584	0.617		

Data are presented as mean ± SD.

BMI, body mass index.

^a^Independent Sample *t*-test.

^b^Paired *t*-test.

*p* value < 0.05 considered significant.

### 3.4. Effect of ellagic acid supplementation on EDSS and general health

Based on the findings of the present study, the average changes of the EDSS index in the ellagic acid group had a significant decrease, the changes of the ellagic acid and control groups were significantly different from each other (−1.06 ± 0.09 vs. 0.04 ± 0.02, *p* < 0.01). The average changes of GHQ and FSS indices in the ellagic acid group had a significant decrease compared to the control group (−5.35 ± 1.94 vs. 0.08 ± 0.04, *p* = 0.032, −1.51 ± 0.42 vs. −0.20 ± 0.08, *p* = 0.028, respectively). The mean changes of PRI index in both ellagic acid and control groups were not significantly different, and the changes of both groups were also insignificant (*p* > 0.05). Also, there were significant differences regarding EDSS, GHQ, and FSS within ellagic acid group (*p* < 0.05) and there were no significant changes in control group at the end of the study compared to their respective baselines (*p* > 0.05; Table 5).

**Table 5:** EDSS, PRI and GHQ status in ellagic acid and control groups in the beginning and at the end of the study.

Variable	Time	Ellagic acid group (*n* = 25)	Control (*n* = 25)	Mean differences (95% CI)	*p*-value^a^	P2^b^
EDSS	Before	2.60 ± 0.38	2.58 ± 0.31	0.02 (−0.17, 0.21)	0.750	
	After	1.54 ± 0.32	2.62 ± 0.29	−1.08 (−1.25, −0.90)	0.001	0.001
	Mean change	−1.06 ± 0.09	0.04 ± 0.02	−1.10 (−1.28, −0.08)	0.001	
	*p*-value^c^	0.001	0.157			
PRI	Before	36.92 ± 5.29	35.83 ± 5.48	1.09 (−1.18, 1.65)	0.882	
	After	36.01 ± 5.32	36.14 ± 5.20	−0.13 (−0.55, 0.29)	0.938	0.927
	Mean change	−0.91 ± 0.94	0.31 ± 1.02	−1.22 (−1.83, 0.54)	0.289	
	*p*-value^c^	0.061	0.096			
GHQ	Before	35.44 ± 6.72	36.16 ± 6.95	−0.72 (−0.94, 0.35)	0.184	
	After	30.09 ± 5.73	36.24 ± 6.83	−6.15 (−8.27, −4.12)	0.027	0.032
	Mean change	−5.35 ± 1.94	0.08 ± 0.04	−5.43 (−7.43, −3.52)	0.032	
	*p*-value^c^	0.018	0.571			
FSS	Before	5.52 ± 0.73	5.82 ± 0.48	−0.30 (−0.67, 0.02)	0.572	
	After	4.01 ± 0.29	5.62 ± 0.55	−1.61 (−2.22, −0.79)	0.041	0.045
	Mean change	−1.51 ± 0.42	−0.20 ± 0.08	−1.71 (−2.39, −0.83)	0.028	
	*p*-value^c^	0.001	0.353			

Data are presented as mean ± SD.

EDSS, Expanded Disability Status Scale; PRI, Pain Rating Index; GHQ, general health questionnaire; FSS, Fatigue Severity Scale.

^a^Independent Sample *t*-test.

^b^General linear model adjusted for baseline differences between groups, age, MS duration.

^c^Paired *t*-test.

*p* value < 0.05 considered significant.

### 3.5. Effect of ellagic acid supplementation on IFNγ, IL-17, IL-4 and TGF-β cytokines and NO

The results indicated that supplementation with ellagic acid caused a significant decrease in the level of IFNγ (−24.52 ± 3.79 vs. −0.05 ± 0.02, *p* < 0.01) and interleukin-17 (−5.37 ± 0.92 vs. 2.04 ± 1.03, *p* < 0.01) in the ellagic acid group and it has caused significant changes between the ellagic acid and control groups. Also, Ellagic acid supplementation led to significant increase in IL-4 levels and there was significant difference between two groups (14.69 ± 0.47 vs. −0.09 ± 0.14, *p* < 0.01). Ellagic acid supplementation led to no significant changes in TGF-β levels in both groups (*p* > 0.05). Besides, our results indicated significant decreasing changes in serum NO (−18.03 ± 1.02 vs. −0.06 ± 0.05, *p* < 0.01) levels after intervention in ellagic acid and control groups. Moreover, there were significant differences regarding IFNγ, IL-17, IL-4 and NO within ellagic acid group (*p* < 0.05) and there were no significant changes in control group at the end of the study compared to their respective baselines (*p* > 0.05). However, there were no significant changes in TGF-β within both ellagic acid and control groups (*p* > 0.05) (Table 6).

**Table 6:** Cytokines and NO status in ellagic acid and control groups in the beginning and at the end of the study.

Variable		Ellagic acid group (*n* = 25)	Control (*n* = 25)	Mean difference (95% CI)	*p*-value^a^	P2^b^
IFNγ (pg/ml)	Before	54.24 ± 3.83	53.14 ± 5.06	1.09 (−1.46, 3.64)	0.394	
	After	29.72 ± 5.89	53.09 ± 5.14	−23.37 (−26.51, −20.22)	0.001	0.001
	Mean change	−24.52 ± 3.79	−0.05 ± 0.02	−24.47 (−28.29, −19.71)	0.001	
	*p*-value^c^	0.001	0.830			
IL-17 (pg/ml)	Before	25.59 ± 5.92	24.48 ± 5.22	1.10 (−2.07, 4.28)	0.489	
	After	19.95 ± 5.99	26.52 ± 8.59	−6.56 (−10.78, −2.34)	0.003	0.003
	Mean change	−5.37 ± 0.92	2.04 ± 1.03	−7.41 (−11.33, −3.47)	0.001	
	*p*-value^c^	0.001	0.065			
IL-4 (pg/ml)	Before	29.32 ± 5.96	29.93 ± 6.80	−0.61 (−4.25, 3.02)	0.735	
	After	44.01 ± 7.87	29.84 ± 6.58	14.16 (9.98, 18.35)	0.001	0.001
	Mean change	14.69 ± 0.47	−0.09 ± 0.14	14.78 (10.04, 18.62)	0.001	
	*p*-value^c^	0.001	0.095			
TGF-β (pg/ml)	Before	1.42 ± 0.05	1.43 ± 0.05	−0.01 (−0.04, 0.01)	0.508	
	After	1.42 ± 0.05	1.43 ± 0.06	−0.01 (−0.04, 0.02)	0.394	0.399
	Mean change	0.00 ± 0.004	−0.02 ± 0.003	0.02 (−0.01, 0.04)	0.639	
	*p*-value^c^	0.285	0.387			
NO (μm)	Before	53.91 ± 1.22	54.05 ± 1.12	−0.14 (−0.09, 0.24)	0.411	
	After	35.88 ± 2.29	53.99 ± 1.18	−18.11 (−22.37, −16.82)	0.001	0.001
	Mean change	−18.03 ± 1.02	−0.06 ± 0.05	−17.97 (−19.21, −15.09)	0.001	
	*p*-value^c^	0.001	0.308			

Data are presented as mean ± SD.

IFNγ, Interferon-gamma; IL-17, Interleukin-17; IL-4, Interleukin-4; TGF-β, Transforming Growth Factor-beta; NO, Nitric Oxide.

^a^Independent Sample *t*-test.

^b^General linear model adjusted for baseline differences between groups, age, MS duration.

^c^Paired *t*-test.

*p* value < 0.05 considered significant.

### 3.6. Effect of ellagic acid supplementation on Tbet, RORγt, and GATA3 gene expression

**Figure 2:**
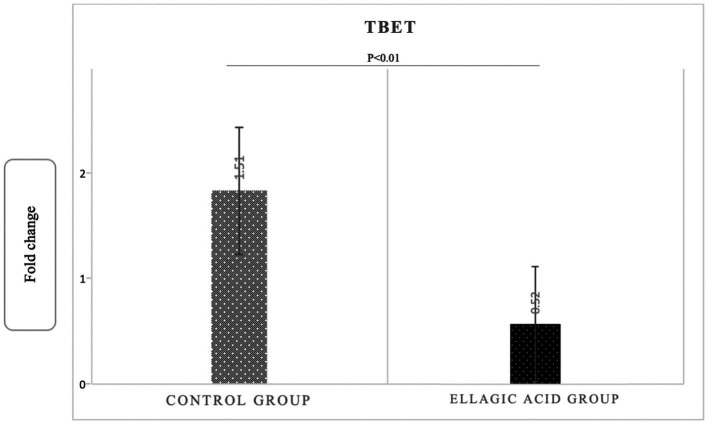
Gene expression of Tbet in ellagic acid and control groups.

**Figure 3:**
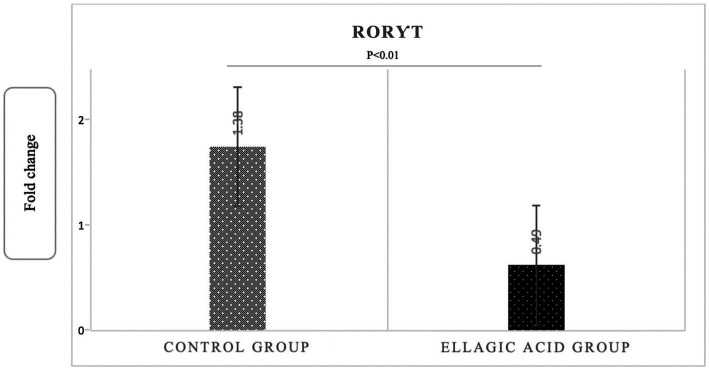
Gene expression of RORγt in ellagic acid and control groups.

**Figure 4:**
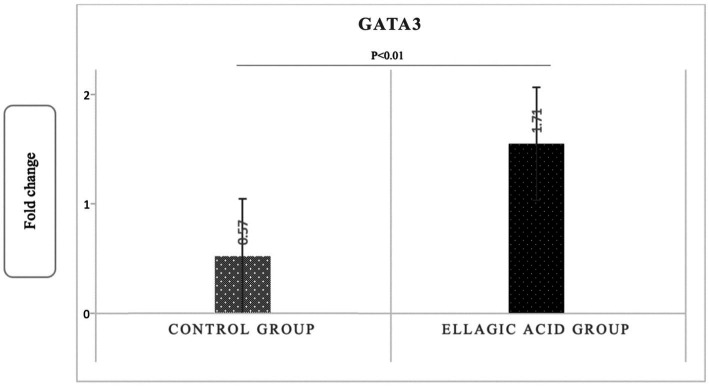
Gene expression of GATA3 in ellagic acid and control groups.

The findings of the present study showed that supplementing with ellagic acid caused a significant decrease in the expression level of tbet and RORγt genes in the group receiving ellagic acid compared to the control group, so that the fold change in the expression of tbet genes in the ellagic acid and control groups was 0.52 ± 0.29 and 1.51 ± 0.18 (*p* < 0.01), respectively. The fold change in the expression of RORγt genes in the ellagic acid and control groups was 0.49 ± 0.18 and 1.38 ± 0.14 (*p* < 0.01), respectively. The Fold change in the expression of GATA3 gene increased significant in ellagic acid group compared to control group (1.71 ± 0.39 vs. 0.27 ± 0.10, *p* < 0.01; Figures 2–4).

## 4. Discussion

The results showed that daily supplementation with 180 milligrams of pure ellagic acid in MS patients decreased the level of inflammatory cytokines, including IL-17 and IFNγ, and increased the serum level of anti-inflammatory cytokines, including IL-4. In addition, ellagic acid supplementation in the present study decreased the tbet and RORγt genes expression and increased the GATA3 gene expression.

MS is an autoimmune disease of the CNS, and the protein components of myelin are the target of the immune system attacks. The role of various immune system factors in the onset of MS has been investigated in several studies (44).

The present study showed that daily intake of 180 mg ellagic acid led to a significant reduction in the serum levels of IFNγ and the tbet gene expression. Noh et al. (45) also reported a decrease in the level of proinflammatory cytokines, including IFNγ, in their study on the ellagic acid effects on dendritic cell maturation. Allam et al. (34), in a study on the potential effect of ellagic acid in the schistosomiasis mansoni treatment in mice, reported a significant decrease in IFNγ with the addition of 600 mg of ellagic acid. In one study, ellagic acid effects at doses of 5, 10, and 20 μg/mL on the immunologic balance of mononuclear cells and colon carcinoma cells were investigated, which at high doses the production of IL-6, TNF-α, IL-1, and IL-10 cytokines suppressed, while showing no effect on IFNγ (46). Some studies found significant increase in IFNγ levels following ellagic acid supplementation. In 2016, Allam et al. conducted a study to investigate the ellagic acid potential effect on the adjuvant induced arthritis (AIA) model in mice and found that supplemenation of 700 mg/kg body weight of ellagic acid let to an increase in IFNγ levels, while TGF-β levels did not change (47). Similarly, Kang et al. (48) reported an increase in IFNγ levels when investigating the effects of ellagic acid on immunologic resistance in transgenic rats carrying hepatitis B virus antigen (48). The reason for this discrepancy with our results is likely due to differences between studies, including differences in the samples studied, the disease studied, the dose of ellagic acid, and the study design. Differentiation of Th1 cells from immature T cells depends on IFNγ and IL-12, which cause expression of T-bet factor *via* activation of the signal transducers STAT1 and STAT4, respectively (49). T-bet causes the production of Th1 cytokines, particularly IFNγ, and in this way enhances the differentiation of Th1 cells. At the same time, T-bet suppresses the differentiation of other subsets of Th cells (50).

The results of studies in the animal model of experimental autoimmune encephalomyelitis (EAE) show that transfer of myelin-specific activated Th1 cells to healthy mice induces EAE in them, and the infiltrated T cells in the CNS mainly produce Th1 cytokines (51). An increase in Th1 cytokines has also been observed in MS patients (52). In studies, an increase in serum levels of the main cytokine of Th1 cells, i.e., IFNγ, was observed in mice with EAE, confirming the pathogenicity of these cells (52). The results of some studies have also shown that disruption of the T-bet gene renders the animals resistant to the induction of EAE (53). Clinical studies have shown that the exacerbation of MS is often associated with the proliferation of myelin-specific Th1 cells in the CSF, and based on pathological observations, the accumulation of Th1 cells and the production of IFNγ in sclerotic plaques are directly related to the demyelination process (54). Moreover, treatment of MS patients with IFNγ increases disease severity, whereas treatment with IFN- neutralising antibodies improves disease progression (17). However, in the present study, ellagic acid was found to decrease Th1 cell activity, as evidenced by a decrease in Tbet gene expression and IFNγ cytokine. Ellagic acid through decreasing the IFNγ, reduces the expression of the death receptor (FAS) on the surface of oligodenocytes and prevents their apoptosis (37, 55). Accordingly, ellagic acid seems to affect Th1 cells, which are responsible for a large proportion of the immunopathologic responses in MS, and to reduce the immunopathologic lesions in MS by preventing the differentiation of naive T cells into Th1 cells, preventing the activation of Th1 cells, and targeting cytokines secreted by Th1 cells or their receptors (56).

Our study showed that daily consumption of 180 mg ellagic acid has immunomodulatory effects on Th17 cells, as evidenced by a significant decrease in Th17 cytokine production. Many studies have investigated the immune system modulating effects of polyphenols, but there are few studies that have investigated the effect of ellagic acid on the immune system. Sanadgol et al. investigated the neuroprotective effect of ellagic acid on acute demyelination by cuprizone with daily supplementation of 40 or 80 mg/kg body weight ellagic acid and observed a significant reduction in IL-17 gene expression (32). Lu et al. showed that pomegranate peel extract exhibited preventive and therapeutic effects in EAE animal models, and this effect is achieved by modulating the gut microbiota, and furthermore, these effects were achieved by inhibiting the filtration of peripheral inflammatory cells into the CNS by reducing the amount of CD4+ IL-17+ and CD4 + IFNγ+ cells (57). In another study, Petrou et al. (58) showed that 6 months of pomegranate seed oil intake in 30 MS patients improved the cognitive characteristics of them. Kiasalari et al. (59) obtained a significant decrease in IL-17 levels following supplementation with 10 or 50 mg/kg body weight ellagic acid in an EAE animal model. Parisi et al. (60) showed in a study that propolis, pomegranate, and grape marc improved RA symptoms and disease severity by lowering the levels of IL-17, IL-1b, and IL-17 stimulating cytokines. Also, in the study on the effect of peel extract of pomegranate on the animal model of EAE and type 1 diabetes, improvement of the symptoms of the disease obtained, and these changes were caused by the inhibition of the filtration of immune cells into the pancreatic islet cells and the reduction of the production of IL-17 and IFNγ (61).

Studies have shown that polyphenols from the tannin family, such as elagitanin, can prevent the production of cytokines by T cells. In addition, these polyphenols can bind to inflammatory cytokines, including IL-17 and IFNγ, or their receptors and prevent their signal transduction, which should be further investigated in future studies. IL-17 is the specific cytokine of Th17 cells. IL-17A induces the production of pro-inflammatory mediators in various cells, all of which demonstrate the pro-inflammatory nature of Th17 cells (62). Th17 differentiation depends on the expression of the RORγt gene. It has been shown that genetic defect of RORγt in mice leads to disruption of Th17 differentiation and protects the mice from induction of EAE disease (63). The results showed that daily supplementation with 180 mg ellagic acid resulted in a significant decrease in levels of IL-17 and RORγt gene expression. In humans, the effects of IL-17 on the process of demyelination of neurons in MS patients are well known, and furthermore, the exacerbation of the disease is related to the increase in the number of Th17 cells in the serum of patients (12). Th17 cells are the first cells to encounter myelin antigens presented by antigen-presenting cells (APC) in the subcranial space. After recognizing the antigen, Th17 cells release several proinflammatory mediators such as IL-17A and create an inflammatory environment that can cause tissue damage in the CNS (64). By stimulating the production of matrix metalloproteinase (MMP) enzymes, IL-17A also causes the destruction of cell junction proteins. IL-17 and ROS also lead to increased expression of endothelial adhesion molecules, resulting in large numbers of inflammatory cells migrating into the CNS (65). Myelin-specific Th17 cells can interact directly with neurons. It seems that ellagic acid by reducing Th17 cells prevents the change of calcium level in the neuron and thus the destruction of neurons. It is also possible that ellagic acid reduces oxidative stress and apoptosis by reducing Th17 cells and IL-17 cytokine levels in oligodendrocytes. By decreasing IL-17 levels, ellagic acid also facilitates the regeneration process of myelin and removes obstacles to the maturation of oligodendrocytes and increases their survival (66, 67).

In the present study, ellagic acid supplementation increased IL-4 levels and GATA3 gene expression. Kang et al. (48) also showed an increase in IL-4 level (48). Rogerio et al. (68), found that a dose of 10 mg/kg ellagic acid inhibited the production of IL-4 in a model of athma. Tanner et al. (69), examined the response of systemic inflammation to ellagic acid in marathon runners and found a significant increase in IL-4 and GATA3 gene expression within 24 h of supplementation. Anderson et al. (70) showed that pure ellagic acid and walnut kernel polyphenols decreased the synthesis of the cytokines IL-13, TNF-a, and increased the production of IL-2, with no effect on IL-4. However, in our study, Th2 cells differentiation increased, as determined by increase in GATA3 expression and IL-4 levels. The increase in the response of Th2 cells to ellagic acid supplementation, which was characterized by the increase in the level of IL-4, is a new phenomenon, and it is likely that it is due to the effect of ellagic acid on the inhibition of Th1 and Th17 responses, because Th1 and Th17 responses are antagonistic with Th2 cells (71, 72). Th2 lymphocytes produce IL-4, IL-5, IL-13, IL-9, and IL-10. GATA-3 inhibits the expression of IFNγ, thereby reducing the Th1 cells response (73). The protective role of Th2 cells dependent on Th1 and Th17 cells has been established; therefore, when the brain is damaged, the immune response tends to favor Th2 cells, which suppress Th1 and Th17 dependent responses and prevent further autoimmune damage in the CNS. Th2 cytokines play a role in reducing the destructive effect of Th1 cells in the EAE (74). A decrease in CNS inflammation and minimal clinical symptoms were also observed in transgenic mice with high expression of GATA3 (leading to a deviation of their immune response toward Th2) after EAE induction. By releasing IL-4, Th2 cells can directly inhibit autoimmune diseases. In the EAE model, the protective effect of Th2 cells has been demonstrated, making it possible to cure EAE by increasing the expression of Th2 cytokines in the brain (75). It appears that ellagic acid, through its effect on naive T cells, induces them to differentiate into Th2 cells, which was observed in the present study by increasing IL-4 levels and GATA3 gene expression. Through the secretion of IL-4, Th2 cells are involved in preventing the free radicals and their propagation through microglial cells (76). Increasing the expression of the GATA3 gene is very important for improving inflammation and disease severity in MS patients. In MS patients, the decrease of Th2 cells and the decrease of the corresponding transcription factor (GATA3) leads to an increase of Th1 cells and their inflammatory cytokines, IFNγ. On this basis, effective treatments also trigger anti-inflammatory responses induced by Th2 cytokines that increase the differentiation of naïve T cells into Th2, for which the transcription factor GATA3 is required. Increasing the expression of the GATA3 gene increases the differentiation of naïve T cells to Th2 and the amount of IL-4, whereupon the number of Th1 cells and inflammatory cytokines and the severity of MS disease decrease. Considering that the absence of phagocytosis of dead and damaged cells leads to disruption of inflammatory processes and remyelination, ellagic acid promotes phagocytosis by M2-type microglia by influencing this process, thus exerting its protective effect (77). Therefore, more attention can be paid to strengthening the activity of Th2 cells in the treatment of MS.

Jha et al. achieved neuroprotective and cognitive improvements in rats suffering from Alzheimer’s disease when they investigated the neuroprotective and cognitive effects of ellagic acid (50 mg/kg), which were confirmed in their study (78).

Another finding in the present study concerned Treg cells activity, which did not change under the influence of ellagic acid and showed no effect on TGF-β levels. Align with our results, Čolić et al. (79), who investigated the immunomodulatory effects of pomegranate peel extract, showed that the Treg cells activity and the levels of TGF-β and IL-10 cytokines decreased in the supernatant of the culture medium. The reason for this finding is probably the increased use of Treg cytokines to downregulate Th1 and Th17 cytokines (79).

### 4.1. Strengths and weaknesses

To our knowledge, our study was the first study to assessed the effect of pure ellagic acid on immunologic parameters, pathogenic genes expression and disease severity in MS. The strength of our study is the ellagic acid purity level (99.9%). In addition, this study discussed the immunological aspects of MS influenced by ellagic acid. However, this study has some limitations. Among them, it was not possible to check the all indicators of oxidative stress in patients in this study. Also, it was not possible to study different doses of ellagic acid. It is suggested that these limitations be addressed in further studies. It would be better if gene expression results were confirmed by measuring protein content, however, in our study, this was not possible due to equipment and budget constraints. It is suggested that in the next studies, a protein measurement method, including Western blotting, should be performed to confirm the gene expression results.

## 5. Conclusion

The present study has shown that supplementation with pure ellagic acid at a dose of 180 mg per day for 12 weeks lowers the levels of the cytokines IFNγ, IL-17, and increases IL-4 so decrease the TH1/Th2 ratio in the group receiving ellagic acid, whereas no significant changes were seen in the placebo group. Supplementation with ellagic acid did not affect TGF-β in any of the study groups. In addition, supplementation with ellagic acid led to the Tbet and RORγt genes expression reduction and GATA3 gene expression increment.

## Data availability statement

The raw data supporting the conclusions of this article will be made available by the authors, without undue reservation.

## Ethics statement

The studies involving humans were approved by Ethics Committee of Iran University of Medical Sciences (Ethics code: IR.IUMS.REC.1399.1000). The studies were conducted in accordance with the local legislation and institutional requirements. The participants provided their written informed consent to participate in this study.

## Author contributions

SJK: hypothesis, writing the draft, laboratory experiments, sampling, and data analysis. NA and A-AD: supervision and edit. FS: edit and review. BA and GH: sampling. MS: data analysis. A-AD and PF: laboratory experiments, KS: data analysis, native edit of manuscript and conceptualization. All authors contributed to the article and approved the submitted version.

## Funding

The funding of the present study was provided by the Iran University of Medical Sciences (Project number: 17787).

## Conflict of interest

The authors declare that the research was conducted in the absence of any commercial or financial relationships that could be construed as a potential conflict of interest.

## Publisher’s note

All claims expressed in this article are solely those of the authors and do not necessarily represent those of their affiliated organizations, or those of the publisher, the editors and the reviewers. Any product that may be evaluated in this article, or claim that may be made by its manufacturer, is not guaranteed or endorsed by the publisher.
